# Modified Incisional Negative Pressure Wound Therapy Increases Seroma Evacuation: An *Ex Vivo* Model

**DOI:** 10.1155/2021/5846724

**Published:** 2021-10-21

**Authors:** Matthias Mehdorn, Boris Jansen-Winkeln

**Affiliations:** Department of Visceral, Transplant, Thoracic and Vascular Surgery, University Hospital of Leipzig, 04103 Leipzig, Germany

## Abstract

**Background:**

Incisional negative wound pressure therapy (iNPWT) is a relatively novel dressing technique with the aim of reducing postoperative wound infections and dehiscence in high-risk wounds after all kinds of surgical procedures. There is a lack of theoretical knowledge about the way those dressing would ameliorate wound healing. One aspect is the reduction of superficial tension, but significant remaining seroma might still cause deep wound infections. The aim of this study was the evaluation of technical modifications of the standard iNPWT dressing to increase seroma evacuation.

**Methods:**

iNPWT dressings were applied on the porcine abdominal wall, and an incremental pressure ramp from 50 to 200 mmHg was performed. The resulting wound pressures were measured using (i) balloon manometry and (ii) esophageal manometry catheter. Seroma evacuation was analyzed with a seroma model. All measurements were performed with (i) standard iNPWT dressing, (ii) wound gauze diverted through the incision, and (iii) placement of suction drain tube into iNPWT.

**Results:**

Due to the modifications of the iNPWT dressing, the vacuum applied by the iNPWT dressing could be transferred into the wound and was not only restricted to superficial layers. More importantly, placement of wound gauzes or suction drain tubes led to complete extraction of wound seroma. The placement of the suction drain tube showed the best combination of increased intrawound pressure as well as seroma evacuation.

**Conclusion:**

Addition of a suction drain tube to the iNPWT dressing leads to an improved function of the iNPWT dressing in our *ex vivo* model.

## 1. Introduction

Negative pressure wound therapy (NPWT) is an established treatment strategy to treat complicated wounds such as dirty-infected primary wounds or wounds showing impaired wound healing due to a superficial surgical site infection with subsequent open wound treatment. It has been shown that the negative pressure increases capillary perfusion of the wound bed [1–3] and is able to absorb wound fluids right away with no need for daily dressing changes. Usually, the NPWT system is placed in the subcutaneous layers of the wound with a sealing foil on the skin.

Over the last decade, evidence was published that presented the possibility to install the NPWT dressing over the closed incision [4], being termed incisional negative pressure wound therapy (iNPWT). Almost from every surgical specialty, there were reports on the benefits of iNPWT. Vascular surgery and orthopedics [5–7] reported decreased rates of surgical site infections (SSI). Furthermore, similar results were published after abdominal wall surgery [8] and after gastrointestinal surgery [9, 10]. Interestingly, the latter cohort study's results could not be confirmed in a randomized controlled trial [11]. The most recent Cochrane collaboration's meta-analysis still gives no clear recommendation on the use of this technique [12] with regard to the reduction of SSI or postoperative seroma. Another meta-analysis, which mainly considered studies in orthopedic surgeries, calculated a seroma reduction of 1.97 ml on day 5 in favor of iNPWT use [13]. Although this result was statistically significant, a difference of nearly 2 ml seroma might not be clinically relevant. A meta-analysis on iNPWT on laparotomies could not show a reduction of seroma formation or wound rupture while still showing an effect on the SSI rate [14]. Some of the most recent randomized trials could not show a reduction of deep postoperative infections in limb surgeries [15] or in mastectomies [16]. The latter study compared iNPWT to standard dressings with active suction drains and noted an increase in postoperative seroma in the iNPWT group.

As data on the benefits of iNPWT remains inhomogeneous, current expert analyses recommend a very careful selection of patients (i.e., those with high-risk wounds or several risk factors for SSI) that might benefit from iNPWT [17].

The biomechanics of iNPWT have been studied by only a few studies. Besides activation of the wound edge perfusion, it has previously been reported that iNPWT mainly works through reduction of the shear stress at the wound edges [18, 19]. Although some have stated reduced postoperative seroma after iNPWT [5], some contradictory studies report the opposite [8, 14, 16]. A porcine study suggests an activation of the lymphatic system by iNPWT to effectively reduce subcutaneous seroma [20].

Our clinical expertise showed several deep wound infections in obese patients with an intact scar but purulent drainage from the bottom of the wound at later stages of the postoperative course after the iNPWT dressing had been removed. Those patients had received wound closure without any additional active suction drains.

Thus, we conducted the following *ex vivo* experimental study with the aim of evaluating possible modifications of a commercially available iNPWT system to improve seroma evacuation from deeper layers of the subcutaneous tissue, using our previously established model of iNPWT [18].

## 2. Methods

As we have previously shown, human and porcine abdominal wall resection samples provided similar results in our experimental setup with the iNPWT systems in place. Therefore, we refrained from experiments with human abdominal wall resectates and limited this series to the porcine abdominal wall. The porcine abdominal wall was bought from the local slaughterhouse. Furthermore, as PREVENA® iNPWT (KCI Acelity, Wiesbaden, Germany) and our self-made epicutaneous VAC systems also showed similar results in the measurements, we performed all experiments with the PREVENA® customizable VAC system on porcine abdominal wall resection samples only.

### 2.1. iNPWT Setup

**Figure 1:**
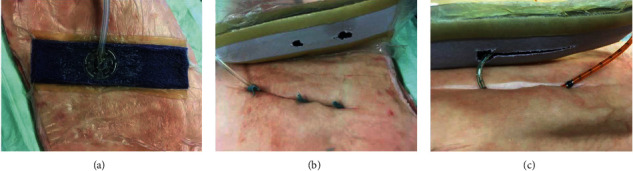
(a) iNPWT installed according to the manufacturer's instructions; (b) wound gauze modification with balloon probe inside the wound; (c) modified iNPWT with suction drain diverted into the iNPWT and with manometry catheter in place.

The PREVENA® iNPWT was installed according to the manufacturer's instructions. Before application of the VAC system, thorough degreasing of the porcine skin was performed. We cut an incision of a length of 10 cm length and 4 cm depth into the porcine abdominal wall After that, the customizable PREVENA was adapted in length with an overlap of at least 2 cm and attached to the skin with the provided adhesive rubber strips and sealed with foil. The Trac Pad® was installed and connected to the VAC device. We used the V.A.C. Ulta and ACTIV.A.C. suction pump (both KCI Acelity, Wiesbaden, Germany). The installed iNPWT dressing is displayed in Figure 1(a).

### 2.2. Test Protocol

After an initial calibration phase with zeroing of the measurement tools, we performed a pressure ramp beginning from 50 mmHg suction up to 200 mmHg in 25 mmHg steps, resulting in 7 definitive measurements. Every step had a duration of 30 sec., and the corresponding pressure was recorded at the end of the step as a certain adaptation of the tissues was seen at the beginning of each step. All measurements were performed as triplicates.

**Figure 2:**
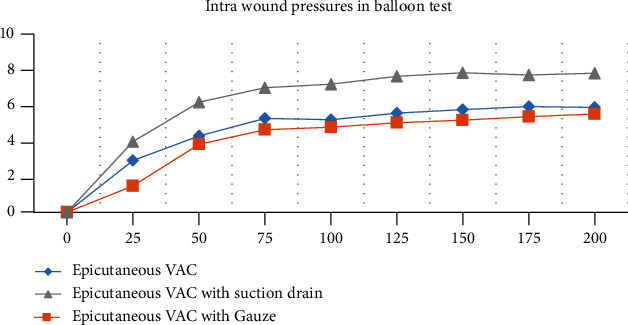
Balloon test of three different types of iNPWT. The *X*-axis indicates negative pressure in mmHg, and the *Y*-axis indicates balloon pressure in mmbar.

Due to the clinical observation of an insufficient seroma evacuation of the iNPWT system, we evaluated two different strategies to ameliorate seroma evacuation: (1) inserting a strip of Cutimed Sorbact® wound gauze (BSN medical, Hamburg, Germany) through the incision (Figure 1(b)) and (2) inserting the tube of a suction drain in the wound bed and diverting it through the incision leading into the V.A.C. foam (Figure 1(c)).

### 2.3. Balloon Test

As previously described, we performed the balloon test using a liquid-filled vinyl balloon [18], which was connected to a manometer (GMH3111, Greisinger Electronics, Regenstauf, Germany). The balloon was placed inside the wound, occupying most of the wound space. After placement of the balloon and installation of the iNPWT dressing, the manometer was zeroed and the pressure ramp initiated. As we performed repetitive measurements, the iNPWT dressing was left in place after each pressure ramp.

### 2.4. Manometry Catheter

The second tool was the 3-channel high-resolution manometry (HRM) catheter for esophageal manometry (Unisensor, Attikon, Switzerland) with the respective real-time analysis software (MM Solar GI HRM, Laborie Europe, Enschede, Netherlands) that was placed diagonally through the incision so that wound pressure was recorded at the bottom and middle and directly subcutaneously. Again, after installation of the dressing and zeroing of the catheter, the pressure was applied.

### 2.5. Seroma Test

For the evaluation of seroma evacuation from the wound bed, we placed an infusion line at the bottom of the wound and diverted it through the subcuticular layers so that an instillation was possible without interfering with the iNPWT system on the skin. As the first step, the volume of the wound was measured by instillation of physiologic saline solution until it was extracted by the iNPWT foam. The measured volume was 5 ml. Therefore, 10 ml of clear saline solution was instilled. and after 2 min of active suction, the dressing was removed and the remaining saline solution measured by syringe aspiration. The test was performed using (1) standard iNPWT dressing, (2) Cutimed® gauze, and (3) suction drain tube, each in triplicates.

### 2.6. Data Collection and Analysis

Data collection and analysis were performed using Microsoft Excel 2016 (Microsoft, Redmond, USA). All results were calculated as means of the triplicate measurements.

## 3. Results

### 3.1. Balloon Test

The data of the balloon test measurements are shown in Figure 2. The iNPWT dressing with an additional suction drain tube inserted via iNPWT foam shows the highest absolute pressure values, but the insertion of a wound gauze did not increase intrawound pressures. The curves reach an asymptotic maximum despite a linear increase in negative pressure.

### 3.2. Manometry Catheter

**Figure 3:**
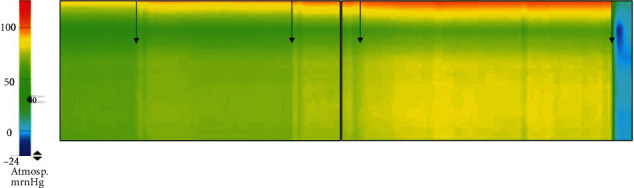
Manometry catheter measurements of the wound. The scale on the left displays the color coding of the measured pressures in mmHg. The images on the right show the pressure within the wound from top to bottom. Arrows indicate a change in negative pressure.

The measurement setup with the diagonally placed manometry catheter showed high-pressure values directly at the skin/surface of the wound and weaker pressures at the bottom of the wound bed (Figure 3). The modifications of the dressing (i.e., wound gauze and suction drain tube) did not lead to significantly different pressure within the wound. Thus, no increase in wound pressure could be detected.

### 3.3. Seroma Test

**Figure 4:**
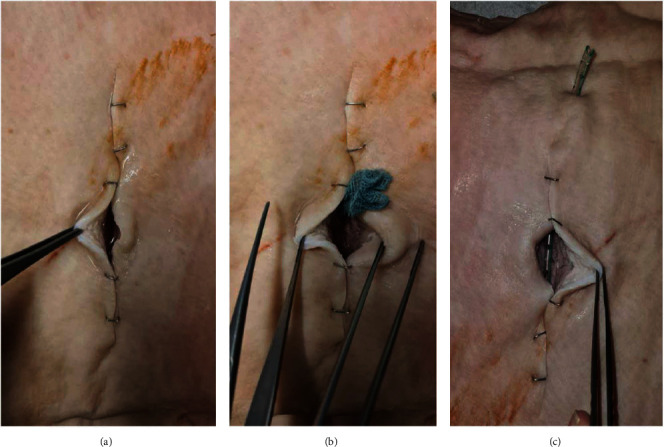
Pictures demonstrate residual seroma in the wound after 2 min of negative pressure in the seroma test: (a) standard PREVENA® with 44% remaining seroma; (b) Cutimed gauze modification with no seroma; (c) suction drain tube modification with no seroma.

First, we performed the instillation test with the regular iNPWT setting. After 2 min of negative pressure, about ±5 ml was still extractable from the wound at each test run, representing a mean 44.4% residual wound fluid. The second modification was the insertion of the wound gauze. After 2 min of negative pressure via iNPWT dressing, a nearly complete evacuation of the fluid could be observed. The same effective fluid evacuation was performed using the suction drain tube that was diverted through the incision. In order to prevent potential wound dehiscence at the site of the diverted drain tube, we used a modified setup: The drain tube was diverted close to the wound edge through the skin but not through the incision itself. Again, almost complete extraction of the wound fluid could be observed. The images of the test series are displayed in Figure 4.

## 4. Discussion

We performed this *ex vivo* experiment of iNPWT in order to find out possible technical modifications of the PREVENA® system to ameliorate wound fluid and seroma extraction from the wound bed since *ex vivo* [18] and *in vivo* [5, 8, 14] data suggest insufficient seroma extraction by iNPWT with subsequent deep wound infections [21]. An explanation for an increased seroma formation in wounds treated with iNPWT might be the wound healing process which is initiated in the epithelial layer. During later stages of the healing process, an invasion of respective cell lines of the subcutaneous tissue occurs. Thus, a top to bottom wound healing occurs. Complete wound closure will be achieved after about eight days [22]. Furthermore, recent studies point out abdominal wall fat thickness as an important predictor of SSI [23], even in wounds after surgeries distant from the abdominal wall [24]. Additionally, it appears that an increased visceral fat area on CT scans also predicts SSI after gastrectomies [25]. This might be attributable to the increased shear stress at the outer abdominal spheres, i.e., fascial layers, according to Laplace's law. This effect is met by the mechanical effect of the iNPWT. Nonetheless, the reduction of wound shear stress at the skin level together with the top to bottom wound healing may lead to entrapment of seroma at deeper wound layers several days after surgery [18, 19]. Therefore, we thought of possibilities on how to improve the negative pressure of the iNPWT dressing at lower wound levels. Traditionally, deeper wounds or large undermining wounds can be closed placing one or more active suction drains to evacuate wound fluids with the aim of preventing wound infection [26] or wound seroma formation [27]. During abdominoplasty, it is considered standard of care to place active suction drains [28] although they might not effectively reduce postoperative seroma [29]. Others have successfully evaluated wound gauzes as a wick in closed incisions to reduce wound infections in dirty wounds [30]. Thus, we evaluated both methods with the aim of finding out if they were able to increase negative pressures at deeper wound layers and if they could potentially reduce remaining seroma inside the wound.

First, we performed the balloon test which represents larger seroma or even hematoma formation in the wound. It was able to display higher intrawound pressures in the setting with a suction drain tube than the regular iNPWT or the wound gauze modification. Therefore, we show an effective increase of the intrawound pressure by insertion of a suction drain tube. In contrast to that, the catheter measurements did not show different pressures at the levels of the wound due to the modification of the dressing. We think that the balloon demands more space within the wound and thus is more sensitive to smaller changes of pressure in the middle of the wound whereas the slim manometry catheter depends on direct contact to tissues to display pressure changes. Nonetheless, we could repetitively show the effect on superficial shear stress, as the pressure was the highest at the skin level ([18]; Figure 3). Our results are coherent with those by Wilkes et al. who reported very slight but significant changes in lateral stress at deeper wound layers in their computed benchtop model [19]. Taken together with the mentioned healing of the wound from top to bottom [22], the use of standard iNPWT suggests improved healing of the cutaneous layer and potential risk of deeper fluid collections.

Besides the aforementioned effects of the modified iNPWT dressings, our data suggests improved fluid extraction from the wound bed by the addition of a suction drain tube that is diverted into the iNPWT foam. Large wounds usually are predisposed to developing wound seroma [14, 29] with the subsequent risk of wound infection in contaminated surgical fields. Improved seroma extraction might reduce wound infection rates or the need for additional therapeutic intervention to treat existing wound seroma.

Until now, relevant large-scale *in vivo* data is lacking on similarly modified iNPWT dressing as we were the first to consider possible modifications based on clinical expertise. Recently, Kitano et al. presented two cases in which they applied a similar iNPWT system with a connected subcutaneous suction drain tube [31]. They termed the apparatus hybrid-iNPWT and used it for hematoma prevention in degloving injuries of two elderly patients. They pointed out the constant negative pressure at the subcutaneous layer as the most important benefit. According to the manufacturer of their suction drain reservoir (J-VAC®), it would supply a maximum of 50 mmHg negative pressure with decreasing forces. Instead, the iNPWT system, due to its electric suction device, allows up to 150 mmHg for infinite time. Therefore, the connection to the device enables constant negative pressure application. This effect might be useful in ventral hernia repair, where large wounds are created during component separation, and thus, high rates of seroma formation may lead to postoperative morbidity [8]. We performed the measurements in linear incisions that are straight, nonwinding cavities. In contrast, degloving injuries represent larger, nonlinear wounds, and the hybrid-iNPWT still seemed to ameliorate wound hematoma formation [31]. Taking together our *ex vivo* and the scarce existing clinical data, it suggests an efficacy on linear as well as wider wounds by modified iNPWT. Furthermore, we believe that our modifications will be applicable not only to the described iNPWT devices (PREVENA® or RENASYS Touch™) but also to other devices with slight technical adaptations.

Our model has several limitations. First and foremost, we provide short-term results in an *ex vivo* setting. Thus, we cannot simulate the real environment of a healing wound with viscous exudates as we used saline solution for seroma simulation. Further, we provide an idea of how to modify the iNPWT dressing to improve several properties. Still, patient safety is also to be considered, as we did not use fixation of the drain tube to prevent its slippage inside the wound or outside the dressing. Additionally, diverting the drain tube into the foam might result in pressure ulcers of the adjacent skin. Hence, further technical considerations of this idea are necessary for safe use. Additionally, clinical data and studies need to prove their efficacy in the reduction of wound infections in clinical use.

## 5. Conclusion

We are the first to demonstrate significantly improved subcutaneous seroma extraction by a modified iNPWT dressing in our *ex vivo* experimental study. The addition of a drain tube that is diverted into the dressing assures that the negative pressure is applied to deeper wound layers, and subsequently, seroma can be extracted more effectively to prevent deep wound infections. Clinical studies are mandatory to prove the described effect in daily routine.

## Data Availability

The data of this study is available from the authors upon reasonable request.
